# In vivo antiviral host transcriptional response to SARS-CoV-2 by viral load, sex, and age

**DOI:** 10.1371/journal.pbio.3000849

**Published:** 2020-09-08

**Authors:** Nicole A. P. Lieberman, Vikas Peddu, Hong Xie, Lasata Shrestha, Meei-Li Huang, Megan C. Mears, Maria N. Cajimat, Dennis A. Bente, Pei-Yong Shi, Francesca Bovier, Pavitra Roychoudhury, Keith R. Jerome, Anne Moscona, Matteo Porotto, Alexander L. Greninger

**Affiliations:** 1 Department of Laboratory Medicine and Pathology, University of Washington School of Medicine, Seattle, Washington, United States of America; 2 Galveston National Laboratory, University of Texas Medical Branch, Galveston, Texas, United States of America; 3 Department of Experimental Pathology, University of Texas Medical Branch, Galveston, Texas, United States of America; 4 Department of Microbiology and Immunology, University of Texas Medical Branch, Galveston, Texas, United States of America; 5 Department of Biochemistry and Molecular Biology, University of Texas Medical Branch, Galveston, Texas, United States of America; 6 Center for Host–Pathogen Interaction, Columbia University Medical Center, New York, New York, United States of America; 7 Vaccine and Infectious Disease Division, Fred Hutchinson Cancer Research Center, Seattle, Washington, United States of America; 8 Department of Pediatrics, Columbia University Medical Center, New York, New York, United States of America; 9 Department of Microbiology & Immunology, Columbia University Medical Center, New York, New York, United States of America; 10 Department of Physiology & Cellular Biophysics, Columbia University Medical Center, New York, New York, United States of America; 11 Department of Experimental Medicine, University of Campania “Luigi Vanvitelli,” Caserta, Italy; New York University School of Medicine, UNITED STATES

## Abstract

Despite limited genomic diversity, severe acute respiratory syndrome coronavirus 2 (SARS-CoV-2) has shown a wide range of clinical manifestations in different patient populations. The mechanisms behind these host differences are still unclear. Here, we examined host response gene expression across infection status, viral load, age, and sex among shotgun RNA sequencing profiles of nasopharyngeal (NP) swabs from 430 individuals with PCR-confirmed SARS-CoV-2 and 54 negative controls. SARS-CoV-2 induced a strong antiviral response with up-regulation of antiviral factors such as *OAS1-3* and *IFIT1-3* and T helper type 1 (Th1) chemokines *CXCL9/10/11*, as well as a reduction in transcription of ribosomal proteins. SARS-CoV-2 culture in human airway epithelial (HAE) cultures replicated the in vivo antiviral host response 7 days post infection, with no induction of interferon-stimulated genes after 3 days. Patient-matched longitudinal specimens (mean elapsed time = 6.3 days) demonstrated reduction in interferon-induced transcription, recovery of transcription of ribosomal proteins, and initiation of wound healing and humoral immune responses. Expression of interferon-responsive genes, including *ACE2*, increased as a function of viral load, while transcripts for B cell–specific proteins and neutrophil chemokines were elevated in patients with lower viral load. Older individuals had reduced expression of the Th1 chemokines *CXCL9/10/11* and their cognate receptor *CXCR3*, as well as *CD8A* and granzyme B, suggesting deficiencies in trafficking and/or function of cytotoxic T cells and natural killer (NK) cells. Relative to females, males had reduced B cell–specific and NK cell–specific transcripts and an increase in inhibitors of nuclear factor kappa-B (NF-κB) signaling, possibly inappropriately throttling antiviral responses. Collectively, our data demonstrate that host responses to SARS-CoV-2 are dependent on viral load and infection time course, with observed differences due to age and sex that may contribute to disease severity.

## Abstract 2

Despite limited genomic diversity, SARS-CoV-2 has shown a wide range of clinical manifestations in different patient populations; the mechanisms behind these host differences are still unclear. This study reveals that SARS-CoV-2 infection induces inflammation by activation of interferon signaling, with differences in host response observed due to sex and age.

## Introduction

The novel coronavirus severe acute respiratory syndrome coronavirus 2 (SARS-CoV-2) that emerged in late 2019 from Wuhan, China, has rapidly spread throughout the world, causing more than 6 million cases and 400,000 deaths globally as of June 2020. Coronavirus disease 2019 (COVID-19) morbidity and mortality has been overwhelmingly concentrated in elderly individuals and those with preexisting comorbidities [1]. In older individuals, immunosenescence and dysregulated antiviral responses due to viral chronic low-grade age-related inflammation may play an important role [2], as has been proposed for influenza [3]. Males are known to be generally more susceptible to infectious disease than females [4], and severe acute respiratory syndrome coronavirus (SARS-CoV)-infected male mice had increased infiltration of inflammatory macrophages into their lungs, leading to a deleterious inflammatory response [5]. Accordingly, systemic inflammatory markers such as neutrophil-to-lymphocyte ratio and C-reactive protein were elevated in men who died of SARS-CoV-2 [6]. However, the mechanisms behind increased mortality among older adults and males with COVID-19 remain speculative.

Entry of SARS-CoV-2 into host cells depends on binding to the receptor angiotensin-converting enzyme 2 (ACE2) [7], expressed at a high level in the nasal epithelium [8], then further induced upon exposure to interferon [9], suggesting a mechanism by which SARS-CoV-2 exploits host antiviral responses. SARS-CoV antagonizes initial viral detection and interferon responses by an as-yet unknown mechanism [10,11]. SARS-CoV-2 may employ similar mechanisms, as infections of bronchial epithelial cells at low multiplicity of infection (MOI) do not result in extensive transcription of interferon-stimulated genes (ISGs) at 24 hours post infection [12]. An important consequence of these observations is that SARS-CoV-2 viral load and transmissibility peaks at the time of symptom onset [13,14]. The temporal relationship between viral load and host gene expression has not been fully explored.

In the United States, diagnostic testing is generally performed on nasopharyngeal (NP) swabs, from which SARS-CoV-2 RNA can be recovered. Shotgun RNA sequencing of this material allows for simultaneous recovery of viral genomes for transmission tracking as well as understanding of in situ host response [15]. Since the first detection of SARS-CoV-2 in the US in Washington State, the University of Washington Virology Laboratory has performed shotgun RNA sequencing to recover more than 1,500 viral genomes as of August 16, 2020, to understand the evolution and molecular epidemiology of the virus [16,17]. Here, we examine host-specific gene expression differences by SARS-CoV-2 infection status, host age, sex, and viral load in NP swabs from 430 SARS-CoV-2-infected individuals and 54 negative controls.

## Results

Between early March and August 16, 2020, the University of Washington Virology Laboratory tested more than 550,000 samples, primarily NP swabs, for infection with SARS-CoV-2 [18]. Thousands of SARS-CoV-2 positive samples, as well as negative controls, have subsequently been metagenomically sequenced, contributing to a detailed understanding of the phylogeny and molecular epidemiology of the virus [16,19]. In this study, we selected a subset of sequenced samples that had sufficient reads pseudoaligned to the human transcriptome (>500,000, median 1.61 × 10^6^) to examine gene expression changes as a result of reverse transcription polymerase chain reaction (RT-PCR)-confirmed SARS-CoV-2 infection. Patient demographics are summarized in Table 1.

**Table 1:** Patient demographics of SARS-CoV-2 positive and negative samples.

			Sex			Age (y)		N1 Ct	
SARS-CoV-2 Status	Viral Load	Total Number	Male	Female	Unknown	Median	Range	Mean	Range
**Positive**		430	176	201	53	54	2–98	21.21	12.32–30.54
	**Low**	99	45	50	4	59	2–96	25.64	24.00–30.54
	**Medium**	206	90	99	17	56	12–98	21.33	19.08–23.99
	**High**	108	41	52	15	52	16–97	16.92	12.32–18.93
	**Unknown**	17	Unk	Unk	17	Unk	Unk	Unk	Unk
**Negative**	**n/a**	54	30	24	0	46.5	12–90	n/a	n/a

**Abbreviations:** Ct, cycle threshold; n/a, not applicable; N1, SARS-CoV-2 nucleocapsid gene region 1; SARS-CoV-2, severe acute respiratory syndrome coronavirus 2; Unk, Unknown

### SARS-CoV-2 infection induces an antiviral, interferon-mediated host response

**Fig 1:**
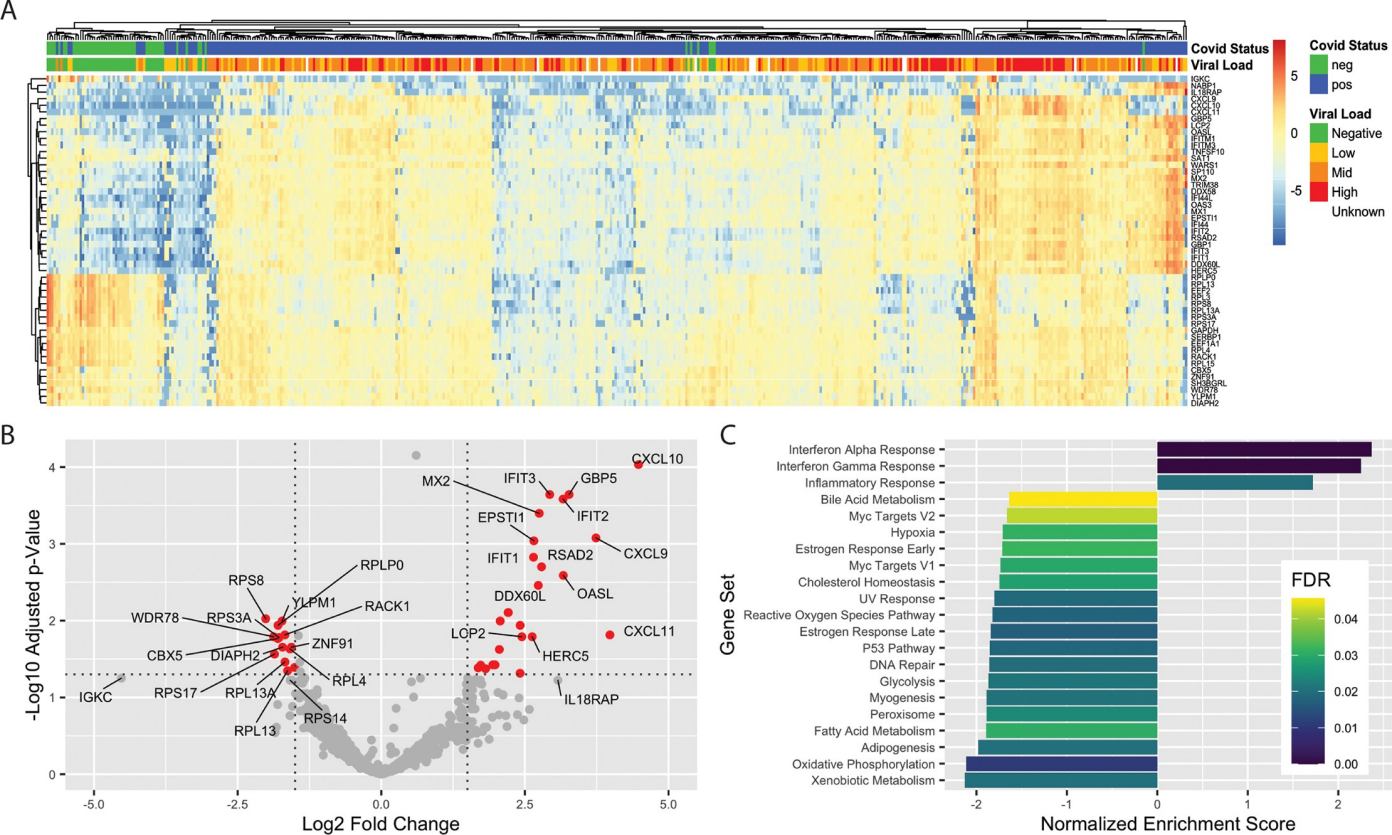
DE genes in SARS-CoV-2 NP swabs. (A) Clustering of samples based on 50 genes with the lowest adjusted *p*-value. Log2 fold changes relative to gene mean are displayed by color. (B) Volcano plot of 15 most up-regulated and 15 most down-regulated genes in SARS-CoV-2 positive samples relative to negative by log2 fold change. Red color indicates genes with log2 fold change > |1.5| and adjusted *p* < 0.05. (C). Significant (FDR < 0.05) pathways affected by SARS-CoV-2 infection identified by GSEA. Raw data available in the GEO Repository, accession GSE152075. DE, differentially expressed; FDR, false discovery rate; GEO, Gene Expression Omnibus; GSEA, Gene Set Enrichment Analysis; NP, nasopharyngeal; SARS-CoV-2, severe acute respiratory syndrome coronavirus 2.

We first characterized the genes most differentially expressed (DE) in the nasopharynx as a result of SARS-CoV-2 infection (*n* = 430 positive, 54 negative). After correcting for batch effects, we found 83 DE genes (*p*_adj_ < 0.1 and absolute log2 fold change > 1) between SARS-CoV-2 positive and negative samples, comprising 41 up-regulated genes and 42 down-regulated genes (S1 Table). Clustering of samples by the 50 most significant DE genes reveals multiple gene expression clusters among SARS-CoV-2 positive samples, while most negative samples cluster together (Fig 1A). Consistent with results from Butler and colleagues [20], SARS-CoV-2 infection induces an interferon-driven antiviral response in the nasopharynx, up-regulating transcripts encoding viral sensors (*IFIT1*, *DDX58*), chemokines that attract effector T cells and natural killer (NK) cells (*CXCL9*, *10*, *11*), and direct inhibitors of viral replication and function (*MX2*, *RSAD2*, *HERC5*), highlighted in Fig 1A and 1B.

To interrogate the global regulatory and signaling programs induced by SARS-CoV-2 infection, we employed Gene Set Enrichment Analysis (GSEA) [21,22] of the 50 Hallmark Gene Sets of the Molecular Signatures Database [23]. Sets with a significant (false discovery rate [FDR] < 0.05) positive enrichment score included Interferon Alpha, Interferon Gamma, and Inflammatory Responses (Fig 1C, S1A Fig). Interestingly, we also found several metabolic pathways negatively enriched, including both Oxidative Phosphorylation and Glycolysis, suggesting a global reduction in production of proteins related to cellular energy production (Fig 1C, S1B Fig). Broad down-regulation of transcripts encoding metabolic machinery may represent either an antiviral response or viral-mediated disruption of host transcripts. We also performed a statistical enrichment test against the Biological Processes Gene Ontology (GO) [24,25]. The most enriched processes (S1C–S1E Fig) are related to either immune responses or translation. In addition to up-regulation of innate antiviral transcripts (Fig 1B, S1D Fig), we also found a consistent down-regulation of transcripts encoding ribosomal proteins (S1E Fig).

### ISGs are differentially expressed as a function of viral load

**Fig 2:**
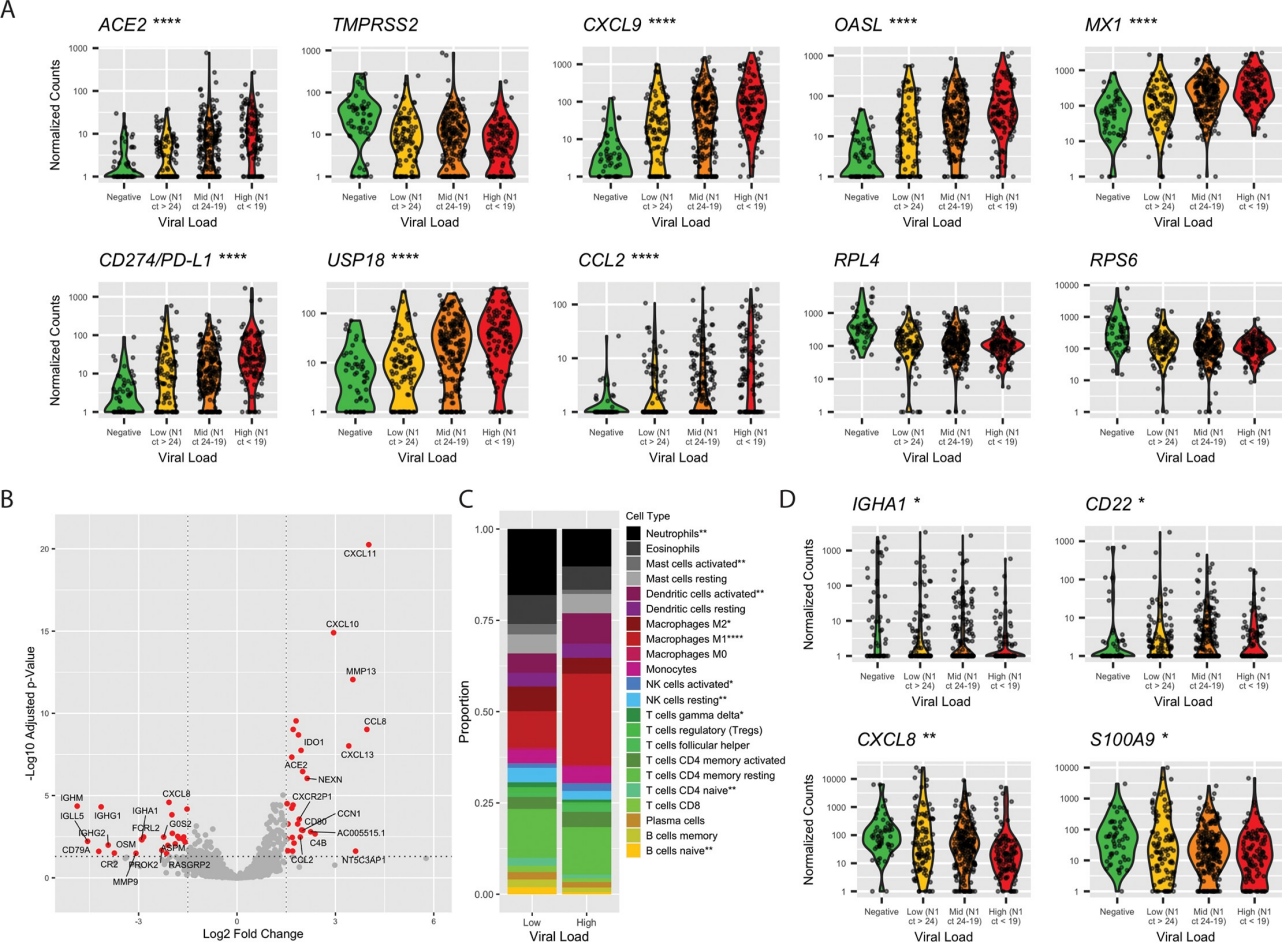
Differences in gene expression by SARS-CoV-2 viral load. (A) Violin plots of select genes by viral load. Statistical significance between low and high viral load calculated by Mann Whitney U test, *p < 0.05, **p < 0.01, ***p < 0.001, ****p < 0.0001. (B) Volcano plot of 15 most up-regulated and 15 most down-regulated genes in SARS-CoV-2 high viral load samples relative to low viral load by log2 fold change. Red color indicates genes with log2 fold change > |1.5| and adjusted *p* < 0.05. (C) Proportion of cell types as a total of all immune cells, by CIBERSORTx. Significant differences in proportion of each cell type determined by *t* test, *p < 0.05, **p < 0.01, ***p < 0.001, ****p < 0.0001. (D) Violin plots of B cell transcripts and neutrophil chemokine transcripts by viral load. Statistical significance between low and high viral load calculated by Mann Whitney U test, *p < 0.05, **p < 0.01, ***p < 0.001, ****p < 0.0001. Raw data available in the GEO Repository, accession GSE152075. Ct, cycle threshold; GEO, Gene Expression Omnibus; N1, SARS-CoV-2 nucleocapsid gene region 1; NK, natural killer; SARS-CoV-2, severe acute respiratory syndrome coronavirus 2; Treg, regulatory T cell.

The SARS-CoV-2 receptor ACE2 is an interferon-regulated gene and is up-regulated in response to SARS-CoV-2 infection [8]. We examined the relationship between viral load—defined by the cycle threshold (Ct) of the SARS-CoV-2 nucleocapsid gene region 1 (N1) target during diagnostic PCR—and *ACE2*. We found that *ACE2* expression was associated with increased viral load: median counts of negative, low viral load (N1 Ct > 24), medium viral load (N1 Ct 24–19), and high viral load (N1 Ct < 19) were 0, 1.93, 3.45, and 7.82, respectively (*p* = 7.46 × 10^−13^, by Kruskal-Wallis one-way ANOVA; Fig 2A). A similar trend was found for other interferon-induced genes, a subset of which is shown in Fig 2A, including those significantly up-regulated in SARS-CoV-2 infection (*CXCL9*, *OASL*, and *MX1*), negative regulators of inflammation (*CD274/PD-L1* and *USP18*), and monocyte chemoattractant protein-1 (*CCL2*) [26]. Conversely, the protease required for viral entry, *TMPRSS2*, was reduced upon viral infection but was not modulated by viral dose, nor were ribosomal proteins (*RPL4*, *RPS6*).

We next specifically examined gene expression differences between high (N1 Ct < 19, *n* = 108) relative to low (N1 Ct > 24, *n* = 99) viral load samples. Fig 2B highlights the 15 most up-regulated and 15 most down-regulated of the 363 total DE genes (adjusted *p* < 0.1, S2 Table). While genes up-regulated in high viral load samples were dominated by proinflammatory and/or interferon-induced factors such as *CXCL9/10*, *IDO1*, and *CD80*, genes with higher expression in low viral load samples included chemokines for neutrophils (*CXCL8*, *S100A9*), and B cell–specific transcripts (*FCRL2*, *IGHG1*, *IGHM*, *IGLL5*, *IGHG2*, *CD22*). Because this suggested differences in immune infiltration as a result of viral load, we performed in silico cell sorting of immune cells using CIBERSORTx [27] and found a higher proportion of transcripts associated with naïve B and T cells, neutrophils, and M2-polarized macrophages in low viral load samples (3.5-, 2.2-, 1.6-, and 1.8-fold increased, respectively), while high viral load samples contained a larger proportion of M1 macrophages, activated NK cells, and activated dendritic cells (DCs) (2.5-, 1.6-, and 1.6-fold up-regulated, respectively; Fig 2C). Levels of transcripts encoding B cell proteins and neutrophil chemokines varied by viral load (Fig 2D).

### Human airway epithelial cells recapitulate the cell-intrinsic antiviral response seen in NP swabs

**Fig 3:**
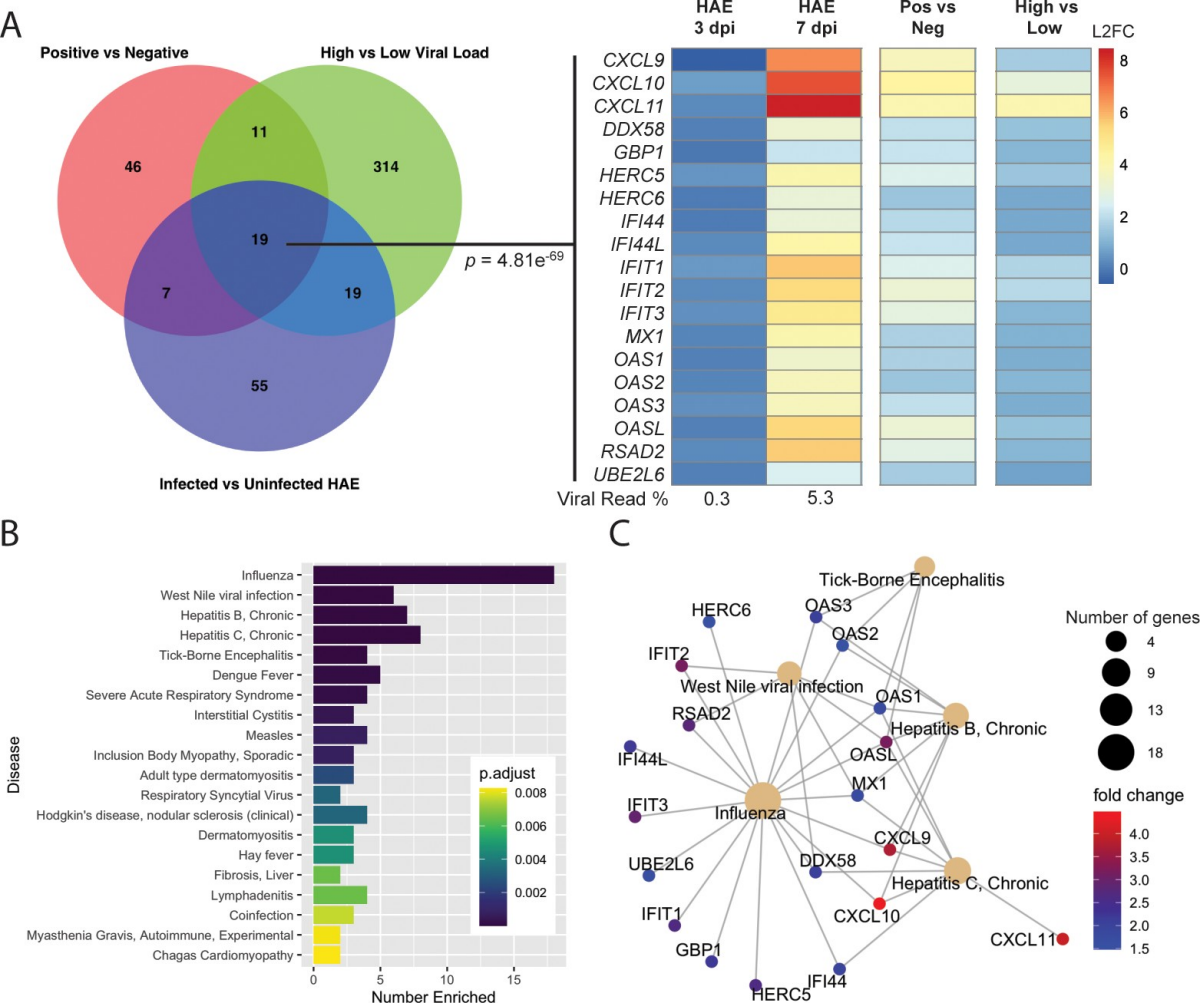
Consensus genes induced upon SARS-CoV-2 expression. (A) Venn diagram of DE genes in SARS-CoV-2-positive versus -negative, high versus low viral load, and top 100 genes with the highest absolute log2 fold change in infected versus uninfected HAE. Consensus set of 19 genes (SuperExact Test, *p* = 4.81 × 10^−69^) DE in all 3 analyses are shown, with log2 fold change values relative to uninfected HAE (for day 3 and day 7 post infection), SARS-CoV-2-negative NP swabs (for SARS-CoV-2-positive NP swabs), or low viral load (for high SARS-CoV-2 viral load samples). SARS-CoV-2 reads at day 3 and 7 post infection were 0.3% and 5.3%, respectively. (B) Top 20 DisGeNET terms for which SARS-CoV-2 cell-intrinsic antiviral response consensus genes are overrepresented. “Number Enriched” is the number of SARS-CoV-2 consensus genes that belong to each disease term. (C) Interaction network of SARS-CoV-2 consensus genes for top 5 most similar diseases identified in panel B. Size of disease node represents the number of genes enriched, and fold change is the log2 fold change seen in SARS-CoV-2-positive versus -negative NP swabs. Raw data available in the GEO Repository, accession GSE154768 (HAE samples) or GSE152075 (NP swabs). DE, differentially expressed; GEO, Gene Expression Omnibus; HAE, human airway epithelial cells; NP, nasopharyngeal; SARS-CoV-2, severe acute respiratory syndrome coronavirus 2.

Detection of differential infiltration of antigen-presenting cells and lymphocytes to the nasopharynx in high versus low viral load samples highlights the role that immune cells play in the host response to SARS-CoV-2. To understand whether in vivo infection could be adequately modeled in vitro, we examined gene expression differences in male human airway epithelial (HAE) cells 3 and 7 days post infection with SARS-CoV-2 and compared the DE genes at day 7 to those from SARS-CoV-2 positive versus negative (Fig 1) and high versus low viral load SARS-CoV-2-positive samples (Fig 2). Although HAE are tracheal/bronchial epithelial cells and thus reside lower in the respiratory tract than the nasopharynx, we nevertheless found a consensus set of 19 up-regulated genes that define cell-intrinsic host antiviral responses to SARS-CoV-2 infection (Fig 3A; *p* = 4.81 × 10^−69^). When this consensus set was tested for statistical enrichment in the DisGeNET [28] of disease ontologies, we found a high degree of overlap with influenza signature genes (Fig 3B), including a number of interferon-induced genes that mediate the acute antiviral response in the respiratory tract (Fig 3C). Notably, in the HAE cells, there was no sign of induction of an interferon response at 3 days post infection in spite of a 10-fold higher infectious dose of virus used and virus making up 0.3% of reads. At 7 days post infection, SARS-CoV-2 constituted 5.3% of reads.

### Longitudinal analysis of patient-matched samples shows reduced viral load and increased wound healing and humoral immune responses over time

**Fig 4:**
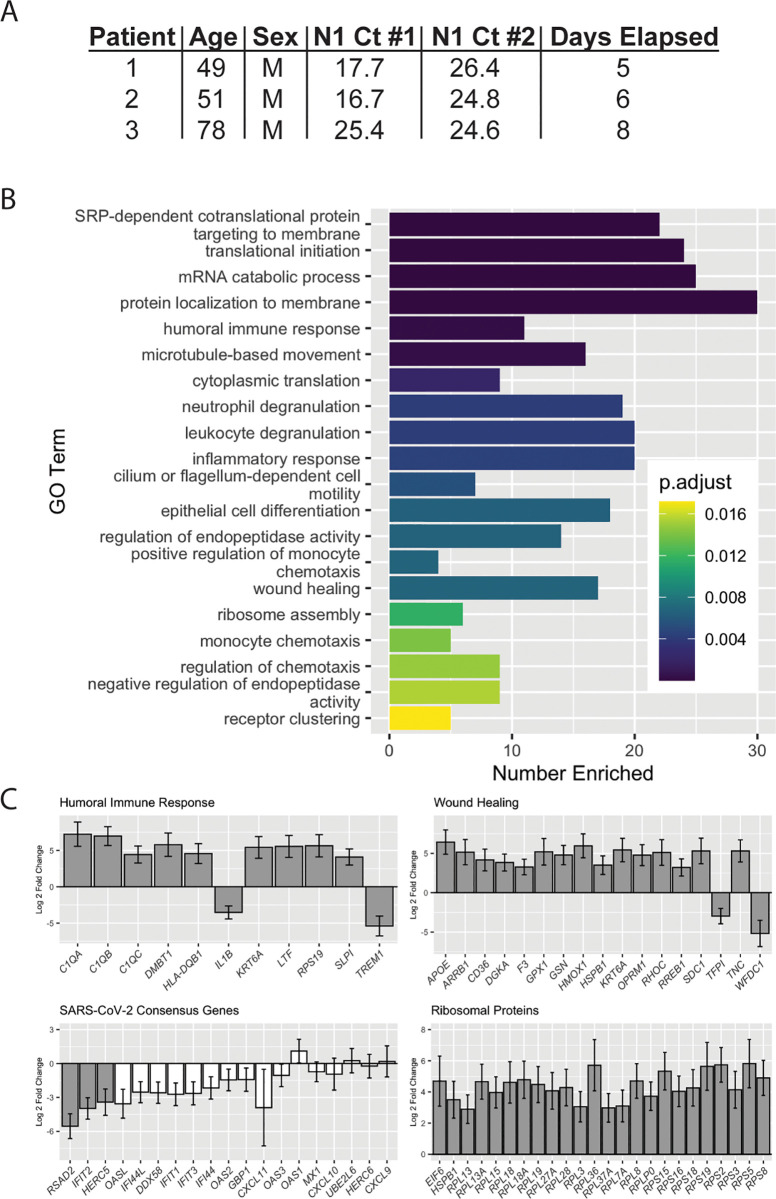
DE genes in patient-matched longitudinal samples. (A) Patient demographics information for longitudinal samples. (B) Top 20 Biological Process GO terms for which longitudinal DE genes are overrepresented. “Number Enriched” is the number of DE genes that belong to each GO term. (C) Log2 fold changes for DE genes in Humoral Immune Response and Wound Healing GO terms, consensus antiviral SARS-CoV-2 genes, and ribosomal proteins. Grey bars: *p*_adj_ < 0.1, white bars: *p*_adj_ > 0.1. Raw data available in the GEO Repository, accession GSE154769. Ct, cycle threshold; DE, differentially expressed; GEO, Gene Expression Omnibus; GO, Gene Ontology; N1, SARS-CoV-2 nucleocapsid gene region 1; SARS-CoV-2, severe acute respiratory syndrome coronavirus 2.

Infection time course may account for the observed differences in immune-related genes in high versus low viral load samples: Patients receiving repeat SARS-CoV-2 testing have a reduced viral load over time [13,29,30]. Although we do not have the ability to tie our data from our large set of positive samples back to the onset of symptoms, we have seen gene expression changes in a small set (*n* = 3) of matched longitudinal samples with a mean elapsed time of 6.3 days between collections and a mean increase in Ct value of 5.29, representing a 39-fold reduction in viral load (Fig 4A). Enriched GO terms include those related to translation and immune regulation (Fig 4B). Notably, in the second sample collected, we saw increases in genes such as *C1QA*, *-B*, and -*C* and *HLA-DQB1* that drive humoral immune responses and those involved in wound healing (*APOE*, *CD36*, *RHOC*), as well as reductions in negative regulators of each process (i.e., *TREM1*, *TFPI*). We also tested the 19 SARS-CoV-2 signature genes (Fig 3A) and found reductions in most at the second collection timepoint, although only *RSAD2*, *IFIT2*, and *HERC5* decreases were statistically significant with 3 samples. We also saw a recovery in the expression of ribosomal proteins over time (Fig 4C). Analysis of data from additional patients with inclusion of more extensive clinical data is currently under investigation in our group.

### Factors affecting T-cell trafficking are reduced in older adults infected with SARS-CoV-2

**Fig 5:**
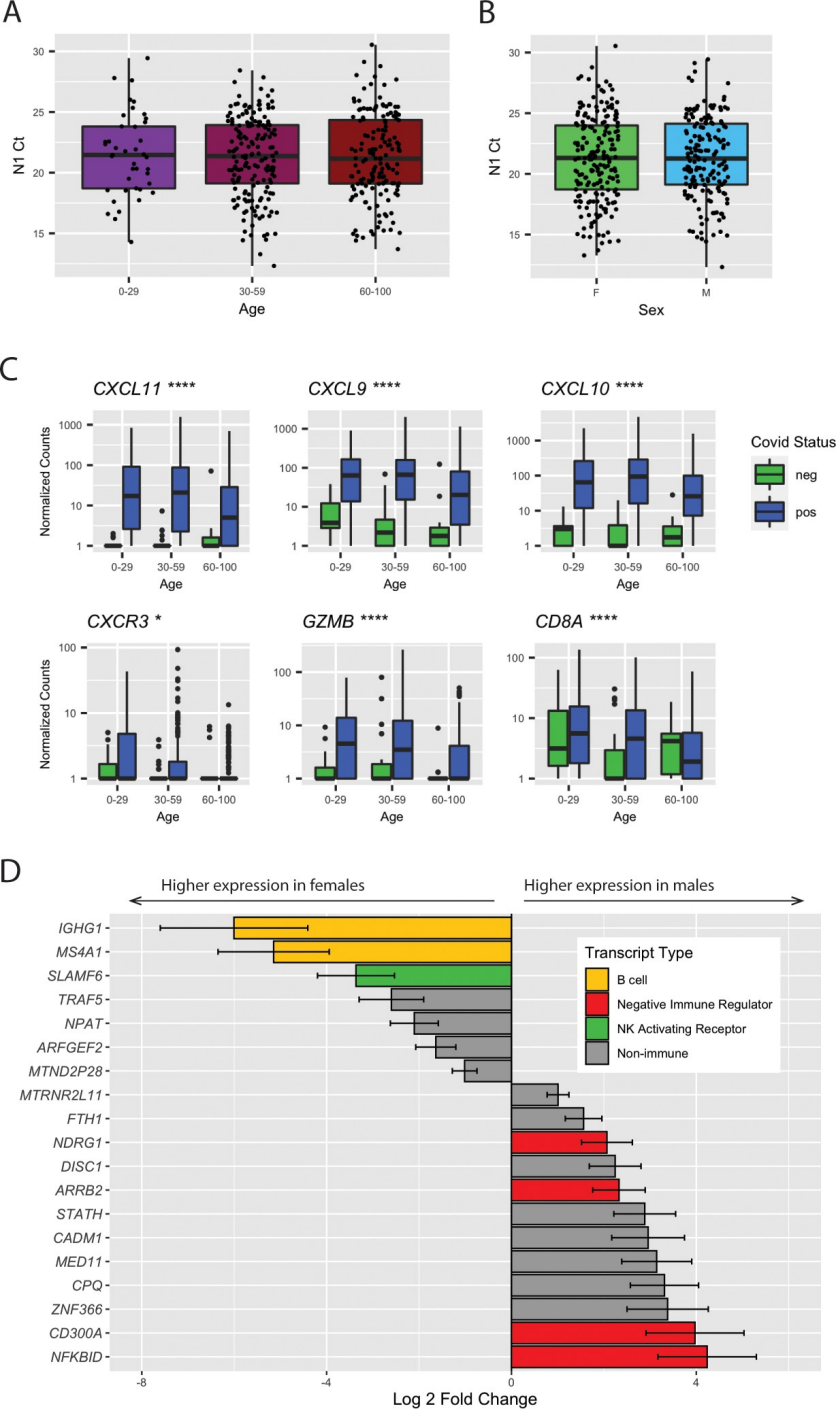
Age and sex cause differences in gene expression upon SARS-CoV-2 infection. (A) N1 Ct values by age group. No significant differences between were observed by Kruskal-Wallis ANOVA. (B) N1 Ct values by sex. No significant difference between groups was observed by *t* test. (C) Gene expression differences by age and viral load. Significance by Mann Whitney U test between SARS-CoV-2-positive samples aged >60 and <60 is shown, *p < 0.05, **p < 0.01, ***p < 0.001, ****p < 0.0001. (D) Sex-modulated DE genes (*p*_adj_ < 0.1) upon SARS-CoV-2 infection. Genes elevated in females are shown as negative log2 fold changes, and those elevated in males as positive log2 fold changes. Raw data available in the GEO Repository, accession GSE152075. Ct, cycle threshold; DE, differentially expressed; GEO, Gene Expression Omnibus; N1, SARS-CoV-2 nucleocapsid gene region 1; NK, natural killer; SARS-CoV-2, severe acute respiratory syndrome coronavirus 2.

Clinically, COVID-19 cases tend to be more severe for older adults and males [1]. No significant difference in N1 Ct was observed based on age or sex (Fig 5A). To understand the differences in host response to SARS-CoV-2 infection, we tested the interaction between infection and age (greater than 60), controlling for non–infection-related age differences with SARS-CoV-2-negative samples. We found only 2 genes altered as a result of the interaction between age and SARS-CoV-2 infection: a 30-fold reduction in production of *CXCL11* (Fig 5B), an interferon-induced chemokine for NK and CD8+ T cells, and a 17-fold reduction in polycomb group factor 6 (*PCGF6*) (S2A Fig), a polycomb repressor complex protein known to play a role in repression of dendritic cell activation [31]. Although we did not find additional genes altered specifically as a result of age in SARS-CoV-2 infection, we did find that *CXCL9* and C*XCL10* are not induced as strongly in SARS-CoV-2-positive patients aged 60 or older. We also found reduced expression of the receptor for *CXCL9/10/11* (*CXCR3*), the apoptosis-inducing factor *GZMB* secreted by NK and T cells, and the effector T-cell marker (*CD8A*). These data suggest that age-related T-cell and NK cell dysfunction [32,33] may play a role in SARS-CoV-2 pathogenesis in older individuals.

### Males and females have differences in immune responses to SARS-CoV-2

We performed a similar analysis to evaluate sex differences in SARS-CoV-2 infection and found a total of 19 genes for which the differences in expression based on sex could be attributed to SARS-CoV-2 infection. S2B Fig highlights the top 10 nonredundant enriched GO categories, most of which are related to immune function. In men, we found down-regulation of B cell–specific transcripts (*IGHG1* and *MSA4A/CD20*), down-regulation of the NK cell–activating receptor *SLAMF6*, and an up-regulation of several inhibitors of NFκB signaling (*NDRG1*, *ARRB2*, *CD300A*, and *NFKBID*) (Fig 5D). The down-regulation of B cell–specific markers suggests differences in lymphocyte composition and/or trafficking in males. Furthermore, the reduction in NK cell–activating receptors and up-regulation of negative regulators of immune effector function—and resultant throttling of effector function—is consistent with a more severe manifestation of COVID-19 in males.

## Discussion

One of the hallmarks of COVID-19 is a dysregulated antiviral immune response. Studies of SARS-CoV, which also employs *ACE2/TMPRSS2*-mediated entry, have demonstrated that infection does not always result in production of interferons in macrophages and dendritic cell [34], and significantly delayed expression of type II or III interferon in lung cells [10]. Moreover, infection of BALB/c mice with SARS-CoV did not result in detectable interferon beta (IFNβ) until 24 hours, at which point viral titers had nearly reached a peak; lung damage resulting from the subsequent massive infiltration of inflammatory macrophages could be abrogated by pre-treatment with type I interferons [11]. Similar viral kinetics have been observed in SARS-CoV-2-infected patients [13,14] and ferrets [12]. Collectively, these results support a common mechanism by which SARS-CoV and SARS-CoV-2 suppress intracellular viral detection and subsequent interferon induction long enough for viral replication to occur.

Our transcriptomic analysis of NP swabs, which were collected primarily after the onset of symptoms, reveals the robust induction of an interferon response by SARS-CoV-2 infection, similar to that observed by Butler and colleagues [20] and consistent with genes regulated by other coronaviruses [35,36]. The highest levels of individual interferon-responsive genes were seen in samples with the highest viral load. Furthermore, although the in silico cell sorting algorithm we employed has not been specifically validated in NP samples, it detected an enrichment of transcripts associated with inflammatory macrophages and activated DCs and NK cells, 3 primary sources of type I and II interferons, in high viral load samples. When repeat swabs were taken from patients with an average 6.3-day time period between sampling, the interferon response had waned, as had viral load, while transcripts associated with an anti-inflammatory, wound-healing response had increased. Notably, in contrast to the robust expression of type I interferon-regulated transcripts in HAE 7 days after infection with SARS-CoV-2, there was limited evidence of induction of an antiviral response after only 3 days, consistent with a SARS-CoV-like functional repression of intrinsic antiviral immunity and interferon signaling. Clearly, high temporal-resolution transcriptional data from both in vitro culture systems and patient-matched longitudinal samples, as well as clinical data for patients, are necessary to understand the early events in viral pathogenesis and immune-mediated resolution of infection, respectively.

COVID-19 patients frequently develop interleukin-6 (IL-6)-driven cytokine release syndrome (CRS), and elevated serum IL-6 correlates with respiratory failure and poor clinical outcomes [37]. Treatment with the IL-6 receptor blocking antibody tocilizumab has effectively treated COVID-19 symptoms in some patients [38]. We did not see a significant difference in expression of *IL-6*, nor of other CRS-associated factors such as *TNF* or *VEGF*, when we analyzed SARS-CoV-2 positive samples relative to negative, nor in high versus low viral load SARS-CoV-2-positive samples. This could be attributed to the nasopharynx not being a particularly sensitive anatomic location to probe markers of systemic inflammation compared to serum or lower respiratory sites; additional studies will be necessary to ascertain how SARS-CoV-2-induced DE of transcripts in the nasopharynx relates to inflammation and pathology in affected organ systems such the lungs, intestinal tract, and circulatory system. Notably, the dissection of interferon responses in the lower respiratory tract will be important in light of recent findings that type III interferons in particular can disrupt healing and barrier function of lung epithelial cells following viral infection [39,40], a possible counterindication to the proposed use of type III interferon as treatment for COVID-19. Finally, our choice to use a large number of samples at relatively low sequencing depth likely reduced our sensitivity to detect differences in low-abundance and short-lived transcripts like cytokines, including interferons.

One of the more striking patterns we observed is the marked down-regulation of transcription of ribosomal proteins upon SARS-CoV-2 infection (Fig 1B) and the recovery of expression during disease progression (Fig 4C). Global inhibition of host transcription is a strategy employed by many viruses via diverse mechanisms such as disrupting transcriptional pre-initiation complex assembly [41,42] or cleavage of TATA-binding protein [43]. Middle East respiratory syndrome-coronavirus (MERS-CoV) and SARS-CoV nsp1 both cause decay of host mRNA [44,45]; in MERS-CoV, host mRNA degradation results from an endonucleolytic function of nsp1 itself [46]. Nsp1 from SARS-CoV and SARS-CoV-2 share 84% amino acid identity, therefore it is likely that SARS-CoV-2 nsp1 can also function directly or indirectly to promote host RNA degradation. Global down-regulation of host transcription may also be driven in part by SARS-CoV-2 ORF6 protein (which binds to the mRNA export factor RAE1 and nuclear pore protein Nup98 in a similar manner as the vesicular stomatitis virus [VSV] M protein) [47] or the ORF7a protein (which binds to proteins involved in ribosomal assembly and nuclear export) [47].

Finally, understanding age- and sex-related differences in responses to SARS-CoV-2 infection is of critical importance as approximately 90% of SARS-CoV-2 deaths in Washington State have been seen in individuals over age 60. Our data show that in individuals over 60, expression of interferon-induced chemokines is reduced, possibly contributing to a reduction in transcripts for cytotoxic T and NK cells. Immune dysfunctions in older individuals are well-characterized [2,3,32,33] and likely contribute to poorer COVID-19 outcomes; results from clinical trials of type I and III interferons in severely ill patients are likely to further define the role of interferon signaling in older adults [48–50].

Differences in immune responses in males and females are due to a variety of factors, including the effects of sex hormones and the X-linked nature of many immune genes [51]. The bias toward expression of B cell transcripts in females in our study is consistent with higher levels of B cells in females regardless of age [52]. Females also tend to have increased inflammation in response to viral infections [4]. The observed increased expression of inhibitors of nuclear factor kappa-B (NF-κB) in males with SARS-CoV-2 may represent either inappropriate throttling of the antiviral immune response or an adaptive mechanism to reduce deleterious inflammation, a hallmark of COVID-19 pathogenesis.

Collectively, we demonstrate induction of an antiviral response characterized by type I and II interferon induction, which wanes with time and is correlated with viral load. We also find evidence of transcriptional repression by SARS-CoV-2. Lastly, we show that differences in immune responses may underlie disparities in outcomes for 2 higher-risk groups, males and the elderly.

## Methods

### Ethics statement

Sequencing of excess clinical samples was approved by the University of Washington Institutional Review Board (STUDY00000408) with a consent waiver.

### Sample collection, RNA extraction, and RT-PCR

NP swabs of patients with suspected SARS-CoV-2 infection were collected in 3 mL viral transport medium (VTM). Total RNA was extracted from 200 or 140 μL of VTM using either the Roche MagNAPure (Roche, Basel, Switzerland) or Qiagen BioRobot (Qiagen, Hilden, Germany) automated platforms, respectively [53]. RT-PCR for the SARS-CoV2 N1 target was performed on the Applied Biosystems 7500 real time PCR instrument [54,55].

### Library preparation and sequencing

Metagenomic next-generation sequencing (mNGS) was performed as previously described [17,56]. Briefly, 18 μL of extracted RNA was treated with Turbo DNAse (ThermoFisher, Waltham, MA). First strand cDNA synthesis was completed using SuperScript IV (ThermoFisher) and random hexamers (Invitrogen, Carlsbad, CA) followed by second strand synthesis by Sequenase version 2.0 (ThermoFisher). The resulting cDNA was purified using either the DNA Clean & Concentrator kit (Zymo, Irvine, California) or 1.6× volumes of AMPure XP beads (Beckman Coulter, Brea, CA). Library preparation was performed using the Nextera XT Kit (Illumina, San Diego, CA). Libraries were cleaned with 0.7× or 0.75× volumes of Ampure beads (Beckman Coulter), quantified using either the Qubit dsDNA HS assay (ThermoFisher) or Quant-iT dsDNA HS assay (ThermoFisher), quality checked by Bioanalyzer or TapeStation (Agilent, Santa Clara, CA), pooled, and sequenced on 1 × 75 bp runs on an Illumina NextSeq or 1 × 101 bp runs on an Illumina NovaSeq.

### Pseudoalignment

Raw FASTQ files were adapter and quality trimmed by Trimmomatic version 0.39 [57] using the call “leading 3 trailing 3 slidingwindow:4:15 minlen 20.” Trimmed reads were pseudoaligned to the Ensembl version 96 human transcriptome using Kallisto version 0.46 [58] assuming an average library size of 300 ± 100 bp. Only samples with more than 500,000 pseudoaligned reads were used for RNA sequencing analysis.

### DE

Pseudoaligned reads were pre-filtered to remove any genes with average expression of less than one read per sample, then normalized, and DE was calculated with the R package DEseq2 version 1.28.1 [59]. Correction for batch effects was incorporated into the design formula and modeling performed using the Wald test with outlier replacement. Results were deemed significant at a Benjamini-Hochberg adjusted *p* < 0.1. Gene expression differences attributable to sex or age were incorporated into the design formula as interaction terms.

### GSEA

GSEA was performed on normalized counts on GSEA software version 4.0.3 [21,22]. Gene ranking was generated with the Signal2Noise metric and analyzed against the mSigDB Hallmarks version 7.1 gene sets [23].

### HAE cultures

The EpiAirway AIR-100 system (MatTek Corporation, Ashland, MA) consists of normal human-derived tracheo/bronchial epithelial cells that have been cultured to form a pseudostratified, highly differentiated mucociliary epithelium closely resembling that of epithelial tissue in vivo. Upon receipt from the manufacturer, HAE cultures were transferred to 6-well plates containing 1.0 mL EpiAirway medium per well (basolateral feeding, with the apical surface remaining exposed to air) and acclimated at 37°C in 5% CO_2_ for 24 hours prior to experimentation.

### Viral growth in HAE

HAE cultures were infected by applying 200 μL of EpiAirway phosphate-buffered saline (MatTeK TEER Buffer) containing 2,000 PFU or 20,000 PFU of infectious clone-derived SARS-CoV-2 expressing a stable mNeonGreen reporter gene (icSARS-CoV-2-mNG) [60] to the apical surface for 90 min at 37°C. At 90 min, the medium containing the inoculum was removed, the apical surface was washed with 200 μL of TEER buffer, and cultures were placed at 37°C. Cultures were fed every other day with 1.0 mL medium via the basolateral side. Media was removed, and cultures were lysed with TRIzol Reagent (ThermoFisher) at 3 days post infection (20,000 PFU challenge) and at 7 days post infection (2,000 PFU challenge). Bam files of viral sequence are deposited in the sequence read archive, NCBI Bioproject PRJNA634194.

### HAE RNA sequencing and analysis

RNA from uninfected and infected HAE was extracted using Direct-zol RNA MicroPrep (Zymo). Libraries were generated using the TruSeq Stranded mRNA kit (Illumina) and 2 × 100 bp paired-end reads sequenced on a Novaseq. Pseudoalignment using Kallisto version 0.44 and DE analysis was performed as earlier.

### Statistics and visualization

All calculations were performed in R version 4.0.0. Statistical enrichment tests of GO [24,25] and DisGeNET [28] pathways were performed in the clusterProfiler R package [61]. Exact test of the intersection of HAE and NP swab consensus genes was performed with the R package SuperExactTest [62]. Images were generated using packages including DOSE [63], ggplot2, pheatmap, and VennDiagram.

## Supporting information

S1 Fig DE gene sets and GO Biological Process terms in SARS-CoV-2 NP swabs. (A) Enrichment plots of gene sets significantly (FDR < 0.05) positively enriched in SARS-CoV-2 samples. (B) Enrichment plots of gene sets significantly (FDR < 0.05) negatively enriched in SARS-CoV-2 samples. (C) Top 20 Biological Process GO terms for which DE genes in SARS-CoV-2 samples are overrepresented. “Number Enriched” is the number of SARS-CoV-2 DE genes that belong to each GO term. (D) Fold change of genes belonging to GO term “defense response to virus.” (E) Fold change of genes belonging to GO term “SRP-dependent cotranslational protein targeting to membrane.” Raw data available in the GEO Repository, accession GSE152075.
(TIF)

S2 Fig Age and sex differences in gene expression upon SARS-CoV-2 expression. (A) Gene expression differences by age and viral load. Significance by Mann Whitney U test between SARS-CoV-2-positive samples aged >60 and <60 is shown, *p < 0.05, **p < 0.01, ***p < 0.001, ****p < 0.0001. (B) Top 10 Biological Process GO terms in which genes defining the male versus female response to virus are overrepresented. Raw data available in the GEO Repository, accession GSE152075.
(TIF)

S1 Table DE genes in SARS-CoV-2-positive samples relative to negative. (CSV)

S2 Table DE genes in SARS-CoV-2 high viral load samples relative to low viral load. (CSV)

## Abbreviations

ACE2: angiotensin-converting enzyme 2
COVID-19: coronavirus disease 2019
CRS: cytokine release syndrome
Ct: cycle threshold
DC: dendritic cell
DE: differentially expressed
FDR: false discovery rate
GO: Gene Ontology
GSEA: Gene Set Enrichment Analysis
HAE: human airway epithelial
IFNβ: interferon beta
IL-6: interleukin-6
ISG: interferon-stimulated gene
MERS-CoV: Middle East respiratory syndrome-coronavirus
mNGS: metagenomic next-generation sequencing
MOI: multiplicity of infection
N1: SARS-CoV-2 nucleocapsid gene region 1
NF-κB: nuclear factor kappa-B
NK: natural killer
NP: nasopharyngeal
RT-PCR: reverse transcription polymerase chain reaction
SARS-CoV: severe acute respiratory syndrome coronavirus
SARS-CoV-2: severe acute respiratory syndrome coronavirus 2
Th1: T helper type 1
Treg: regulatory T cell
VSV: vesicular stomatitis virus
VTM: viral transport medium
