# Supplementary figures and images for: In vivo antiviral host transcriptional response to SARS-CoV-2 by viral load, sex, and age
Source: PLoS Biol. 2020 Sep 8;18(9):e3000849. doi: 10.1371/journal.pbio.3000849 (PMC7478592; doi:10.1371/journal.pbio.3000849)

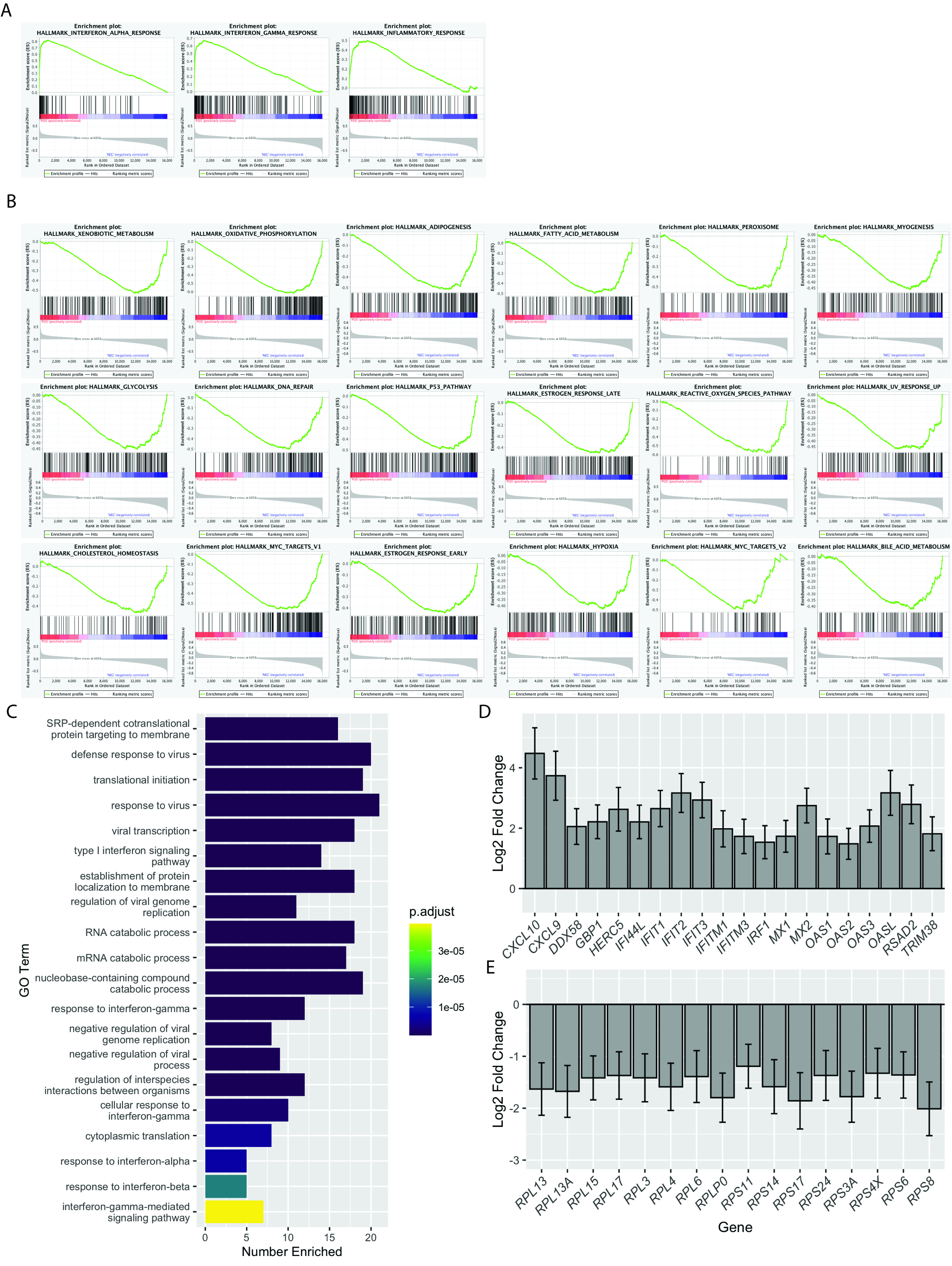

Supplement: S1 Fig — (A) Enrichment plots of gene sets significantly (FDR < 0.05) positively enriched in SARS-CoV-2 samples. (B) Enrichment plots of gene sets significantly (FDR < 0.05) negatively enriched in SARS-CoV-2 samples. (C) Top 20 Biological Process GO terms for which DE genes in SARS-CoV-2 samples are overrepresented. “Number Enriched” is the number of SARS-CoV-2 DE genes that belong to each GO term. (D) Fold change of genes belonging to GO term “defense response to virus.” (E) Fold change of genes belonging to GO term “SRP-dependent cotranslational protein targeting to membrane.” Raw data available in the GEO Repository, accession GSE152075. (TIF) [file pbio.3000849.s001.tif]

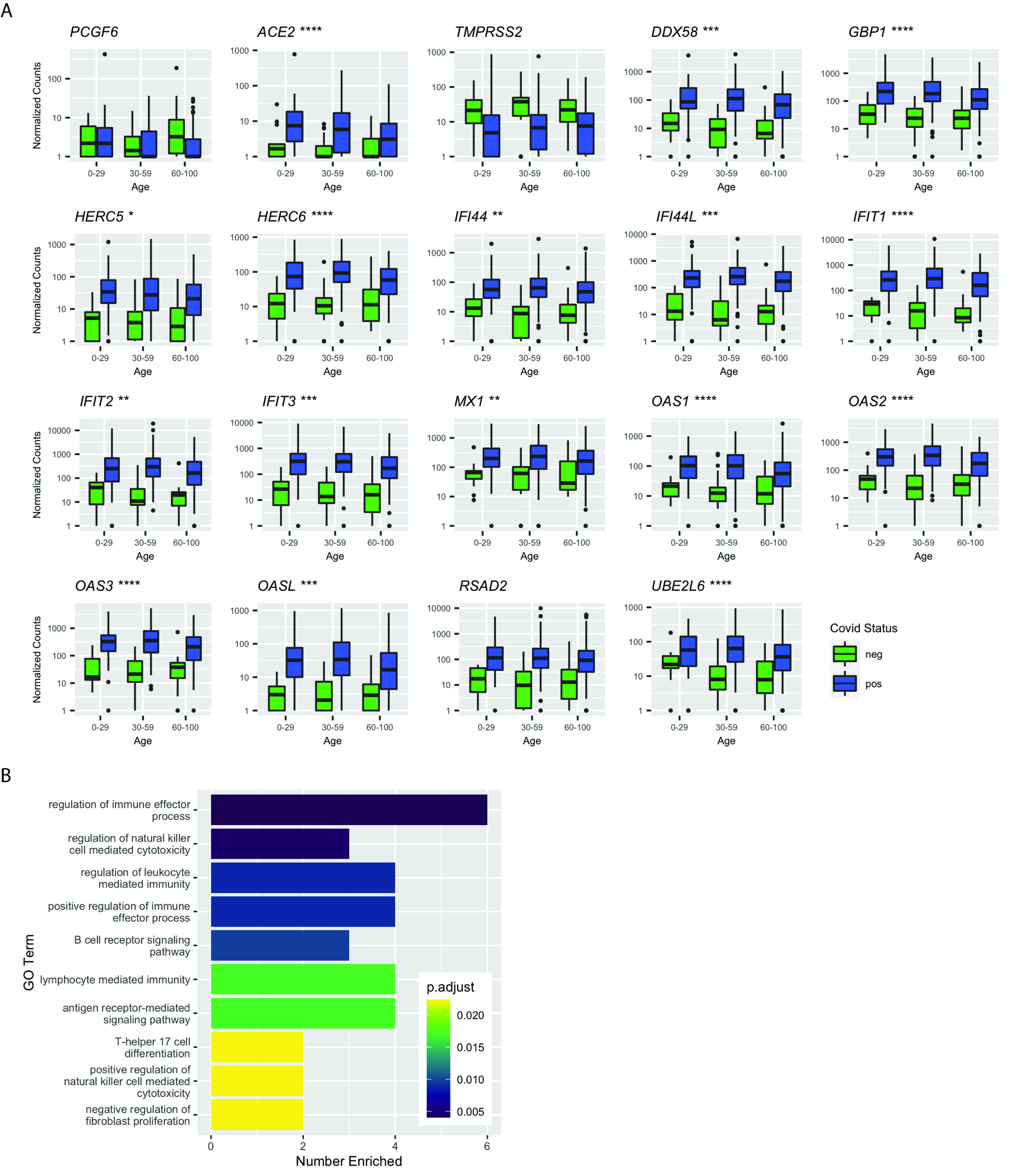

Supplement: S2 Fig — (A) Gene expression differences by age and viral load. Significance by Mann Whitney U test between SARS-CoV-2-positive samples aged >60 and <60 is shown, *p < 0.05, **p < 0.01, ***p < 0.001, ****p < 0.0001. (B) Top 10 Biological Process GO terms in which genes defining the male versus female response to virus are overrepresented. Raw data available in the GEO Repository, accession GSE152075. (TIF) [file pbio.3000849.s002.tif]
